# Fiberoptic Endoscopy Evaluation of Swallowing (FEES) Findings Associated with High Pneumonia Risk in a Cohort of Patients at Risk of Dysphagia

**DOI:** 10.1007/s00455-024-10727-w

**Published:** 2024-07-03

**Authors:** Luis F. Giraldo-Cadavid, Diego Insignares, Valentina Velasco, Natalia Londoño, Ana María Galvis, María Leonor Rengifo, Alirio R. Bastidas-Goyes

**Affiliations:** 1 Department of Epidemiology and Biostatistics, Facultad de Medicina de la Universidad de La Sabana, Chia, Cundinamarca, Colombia; 2 Interventional Pulmonology Division, Fundación Neumológica Colombiana, Bogotá DC, Colombia; 3 Departments of Internal Medicine and Pulmonary Medicine, Facultad de Medicina de la Universidad de La Sabana, Campus Puente del Común, Autopista norte de Bogotá Km 7, Chía, Cundinamarca, Colombia; 4 Department of Rehabilitation Medicine, Facultad de Medicina de la Universidad de La Sabana and Clínica Universidad de La Sabana, Chía, Colombia; 5 Department of Rehabilitation Medicine, Clínica Universidad de La Sabana, Chía, Colombia

**Keywords:** Deglutition, Deglutition disorders, Dysphagia, Respiratory aspiration, Pneumonia, Fiberoptic endoscopic evaluation of swallowing (FEES)

## Abstract

Aspiration detected in the fiberoptic endoscopy evaluation of swallowing (FEES) has been inconsistently associated with pneumonia, with no evidence of the risk of pneumonia from other alterations in swallowing safety detected in FEES. We conducted a dynamic, ambidirectional cohort study involving 148 subjects at risk of dysphagia in a tertiary university hospital. Our aim was to determine the risk of pneumonia attributed to alterations in swallowing safety detected during FEES. We used multivariate negative binomial regression models to adjust for potential confounders. The incidence density rate (IR) of pneumonia in patients with tracheal aspiration of any consistency was 26.6/100 people-years (RR 7.25; 95% CI: 3.50–14.98; *P* < 0.001). The IR was 19.7/100 people-years (RR 7.85; 95% CI: 3.34–18.47; *P* < 0.001) in those with laryngeal penetration of any consistency and 18.1/100 people-years (RR 6.24; 95% CI: 2.58–15.09; *P* < 0.001) in those with pharyngeal residue of any consistency. When adjusted for aspiration, the association of residue and penetration with pneumonia disappeared, suggesting that their risk of pneumonia is dependent on the presence of aspiration and that only aspiration is independently associated with pneumonia. This increased risk of pneumonia was significant in uni- and multivariate negative binomial regression models. We found an independently increased risk of pneumonia among patients with dysphagia and aspiration detected during FEES. Alterations in the oral and pharyngeal phases of swallowing, without aspiration, did not increase the risk of pneumonia.

**Supplementary Information:**

The online version contains supplementary material available at 10.1007/s00455-024-10727-w.

## Introduction

Dysphagia is associated with an increased risk of malnutrition, electrolyte imbalance, and pneumonia [1, 2]. Aspiration pneumonia has a mortality rate higher than 20% [3–5], generating a high burden on society and increased health-care costs [6, 7]. Fiberoptic endoscopy evaluation of swallowing (FEES) and videofluoroscopic evaluation of swallowing (VFSS) are considered the gold standard tests to evaluate oropharyngeal dysphagia [8, 9].

Both FEES and VFSS findings are related to the efficiency and safety of swallowing. Alterations in swallowing safety include aspiration of food below the vocal cords, penetration of food into the laryngeal vestibule, and persistence of food residue in the pharynx after swallowing [9, 10]. Alterations of swallowing efficiency or function refer to delays in bolus transit from the mouth to the stomach or other alterations in the deglutition function not necessarily compromising swallowing safety [11].

Experts in diagnostic test research advocate a phased approach to evaluating clinical tests, mirroring the 4-phase model used for therapeutic interventions [12, 13]. Such phases include test development, reliability assessment (including inter- and intra-rater reliability), diagnostic accuracy, prognostic capacity for outcomes, and clinical effectiveness in decision-making [12, 13]. Prior research has explored FEES’s reliability [14–16] and accuracy [17] as a diagnostic test. Regarding the prognostic capacity of alterations in swallowing safety detected during FEES and VFSS, aspiration detected in VFSS has been associated with a significant increase in the risk of pneumonia [18–20]. Aspiration detected in FEES presents an annual risk of pneumonia higher than 20% [21–23], potentially increasing the risk by up to three times; however, this evidence is still limited due to the poor control of confounding bias in most studies [24]. Penetration detected in VFSS has also been related to an increased risk of pneumonia [20]. However, the presence of penetration in FEES has not been associated with pneumonia, and the evidence supporting the association between penetration and pneumonia in VFSS is still weak [20, 25, 26]. Similarly, there are few studies on the risk of pneumonia in patients with pharyngeal residue [25, 26].

Therefore, the evidence on the prognostic capacity of FEES findings is scarce or limited by poor control of confounding bias [24], and extrapolating the predictive capacity for dysphagia outcomes from VFSS to FEES would be inappropriate, considering that each diagnostic test requires its own validation and the results of a 2017 meta-analysis comparing FEES and VFSS, which found FEES slightly more sensitive in detecting aspiration, penetration, and residue [17]. Furthermore, it is unclear whether alterations in swallowing safety, other than aspiration, independently increase the risk of pneumonia or if the presence of aspiration is necessary for pneumonia to occur.

Considering the high risk of complications associated with pneumonia in patients with muscular or neurological disorders and other causes of functional oropharyngeal dysphagia combined with the high costs associated with the treatment of this condition, it is crucial to detect high-risk subgroups for pneumonia in order to establish preventive measures [6, 7, 27]. For this reason, we decided to conduct a cohort study to evaluate which of the swallowing alterations, particularly aspiration, penetration, and residue detected in FEES, are associated with an increased risk of pneumonia.

## Materials and Methods

We performed an ambidirectional cohort study involving 148 subjects consecutively recruited over a six-year period from an outpatient rehabilitation service at a tertiary care university hospital. The study aimed to explore the relationship between alterations in swallowing safety (penetration, aspiration, or residue) as identified by FEES and the incidence of pneumonia. Subjects with muscular or neurological involvement at risk of functional oropharyngeal dysphagia were included. The exposed cohort was constituted by those subjects with alterations in swallowing safety detected in FEES and the unexposed cohort included subjects without such alterations.

The exclusion criteria were subjects with mechanical dysphagia (tumors or anatomical abnormalities of the upper aero-digestive tract), active cancer, cancer chemotherapy, pregnancy, and asplenia.

Pneumonia was defined as the presence of the symptoms and signs of acute lower respiratory infection (cough, purulent sputum, fever, tachypnea, tachycardia, leukocytosis, or leukopenia), and the presence of infiltrates on a chest radiograph [28, 29].

Socioeconomic level in Colombia is classified according to the place of residence and the payment of public services. Low socioeconomic level is those classified as 1 and 2, medium socioeconomic level to levels 3 and 4, and high socioeconomic level to levels 5 and 6.

### Evaluation of Swallowing

All subjects received a clinical evaluation of swallowing that included a search for signs and symptoms of oropharyngeal dysphagia and an assessment of head and neck muscle function. Further details of the clinical evaluation are included in the supplementary appendix. Subjects were considered at risk of oropharyngeal dysphagia when one or more of the following abnormalities were present: symptoms of pharyngeal dysphagia; any compromise of gag reflex; a gastrostomy, jejunostomy, or nasoenteral feeding tube; head or neck muscle compromise; or an orotracheal or tracheostomy tube for more than 14 days. When the clinical evaluation of swallowing was completely normal, a FEES was not performed. Subjects underwent a FEES in case of any abnormality in the clinical evaluation of swallowing or oropharyngeal muscle motor compromise.

The FEES studies were conducted by a pulmonologist with more than 2 years’ experience and 50 completed FEES procedures along with a speech language pathologist. The studies were done with the subjects in a seated position, without anesthetics, and using a flexible fiberoptic endoscope with an outer diameter of 4.1 mm connected to a video system (Olympus LF-GP, Center Valley, PA, USA). The endoscope was lubricated with water-soluble gel, introduced through one of the nostrils, and advanced into the pharynx. The examination included an anatomical and functional exploration of the upper aero-digestive tract, including vocal cord motility. Subsequently, the tip of the endoscope was placed at the velopharynx. A first swallow without food was conducted to evaluate the pharyngeal contraction and the elevation of the larynx. The evaluation of swallowing continued by observing the bolus transport of green-colored foods of different consistencies from the back of the oral cavity to the esophagus and searching for any abnormality in deglutition.

The FEES unit, connected to the video equipment, recorded the procedures. These recordings were then reviewed in real-time and, when necessary, in slow motion after the examination. Further details of the FEES protocol can be found elsewhere [30].

An association with pneumonia was explored for the following alterations of swallowing safety as detected in FEES: residue, penetration, and aspiration. Residue consisted of the persistence of material in the pharynx (pharyngeal walls, valleculae, epiglottis, pyriform sinuses, and base of the tongue) after swallowing, penetration consisted of the entrance of material into the laryngeal vestibule, and aspiration consisted of the passage of material into the trachea below the vocal cords [30]. Given the existing literature validating the intra- and inter-rater reliability [14–16] and accuracy [17] of the FEES findings we investigated and our study’s focus on other aspects, such as the prognostic capacity of FEES for predicting the occurrence of pneumonia, we did not include measurements of intra- and inter-rater reliability in our study [14, 15].

### Follow-up

The follow-up for the 148 patients spanned five years, comprising two phases. Phase I, the retrospective phase, occurred between the onset of dysphagia (e.g., stroke, brain trauma, or central nervous system infection causing dysphagia) and the FEES. For subjects with a completely normal clinical examination of swallowing, who did not require FEES, this time was between the event causing disability requiring referral to our rehabilitation program and the clinical examination of swallowing. These patients with normal swallowing were included in the unexposed cohort for comparison with the exposed cohort (patients having alterations in swallowing safety detected by FEES). We included this retrospective phase of follow-up in order to analyze the period in which subjects did not receive swallowing rehabilitation, because at the time dysphagia was diagnosed, the subjects were admitted to a standardized protocol aiming to prevent respiratory complications and rehabilitate the swallowing process, which could weak the relationship between dysphagia and pneumonia. Phase II of the follow-up (prospective phase) was between the FEES or the normal clinical examination of swallowing and the end of the study. We determined the frequency of pneumonia during both phases of follow-up. The mean follow-up duration for Phase I was 1.8 years, and for Phase II was 3.7 years, resulting in a total mean follow-up of 5.5 years. Subjects underwent follow-up FEES when indicated by changes in the clinical evaluation of swallowing.

### Ethical Considerations

The study was performed according to the international standards of the Helsinki Declaration and national regulations. It was approved by the local Institutional Review Board and all subjects provided informed consent to participate in the study.

### Statistical Analysis

Qualitative variables were summarized as frequencies and percentages, and quantitative variables were summarized as means ± standard deviation (SD) if normally distributed, or median and interquartile range (25th–75th percentile) if non-normally distributed. The number of pneumonia episodes were analyzed in terms of incidence density rate (IR), namely the number of pneumonia episodes over the sum of the person-time of the people at risk (number of cases/100 people-years).

We implemented strategies to reduce selection bias, to control observer bias, and to prevent information loss, all of which are detailed in the supplementary appendix.

**Fig. 1:**
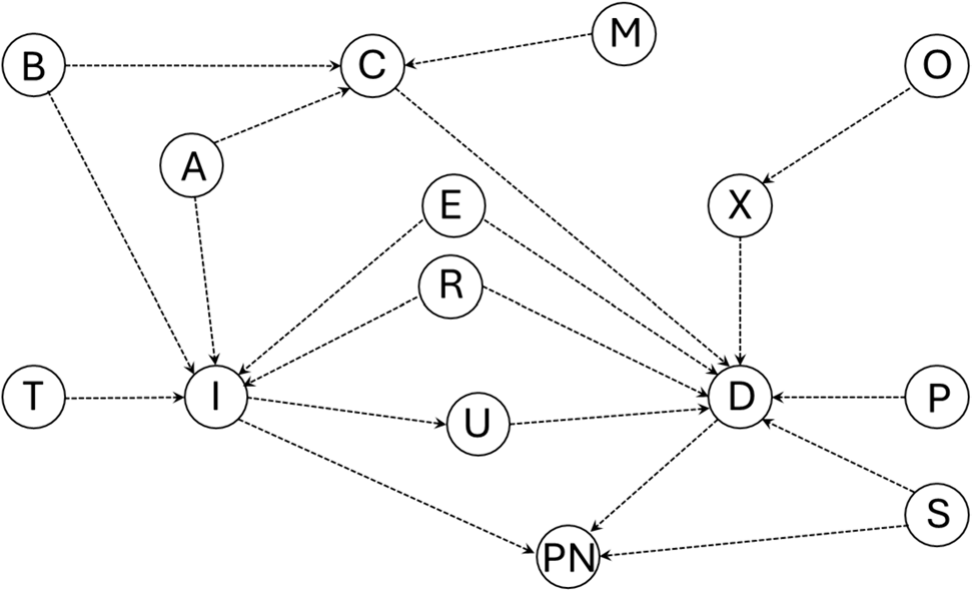
Causal pathways between comorbidities and pneumonia. Notes: A: age, sex, malnutrition, alcohol consumption; B: chronic obstructive pulmonary disease (COPD), asthma, diabetes; C: compromised pharyngeal function; D: dysphagia; E: antacids; I: immunosuppression; M: central nervous system depressant drugs, cerebrovascular disease, muscular and neuromuscular diseases, compromised state of consciousness, dementia, central nervous system cancer, head and neck cancer, degenerative neurological diseases; O: medications that decrease saliva production, hypertension; P: gastroesophageal reflux, esophageal dysmotility, upper gastrointestinal tract surgery, congestive heart failure; PN: pneumonia; R: inhaled corticosteroid, systemic corticosteroid; S: oral hygiene, smoking; T: cirrhosis; U: infections in the oropharynx and lacerations in the mouth, throat and esophagus; X: xerostomia

The independent variables included penetration, aspiration, and pharyngeal residue; the dependent variable was the number of episodes of pneumonia. To control confusion bias, we built multivariate logistic and negative binomial regression models that included the alterations of swallowing safety and the variables that could affect the association between dysphagia and pneumonia selected by biological plausibility and the drawing of directed acyclic graphs (DAG) (Fig. 1). The variables for the bivariate analysis selected by DAG were age at time of event, sex [31], smoking [32], chronic respiratory diseases (COPD, asthma, and other respiratory diseases) [33, 34], antacids [35], proton-pump inhibitors, oral hygiene frequency per day [36, 37], use of inhaled and systemic corticosteroids [38], diabetes, congestive heart failure, use of atypical and typical antipsychotics [39], high alcohol consumption defined as more than 3 drinks per day and/or more than 6 drinks per occasion in women or more than 8 drinks per occasion in men [40, 41], and immunosuppression [42]. The variables associated with the outcome (pneumonia) and the exposure (alterations of swallowing safety) in the univariate analysis with *P* < 0.25 at two tails and that fulfilled the criteria to be potential confounders according to DAGs (potential association with the exposure and outcome and not being intermediate variables in the causal path from the exposure to the outcome were entered into the multivariate model, and those with statistical significance in the multivariate model or those that changed the regression coefficient of the exposure under study by more than 10% were retained. Statistical significance was set at *P* < 0.05 (two-tailed).

**Fig. 2:**
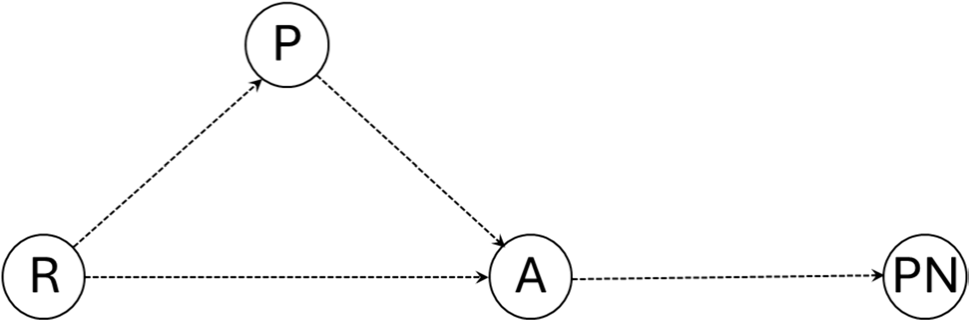
Causal pathway between alterations in the safety of swallowing detected in the FEES and pneumonia. Notes: R: pharyngeal residue; P: penetration; A: aspiration; PN: pneumonia

To investigate whether the association between penetration, residue, and the occurrence of pneumonia was independent or mediated by aspiration, we constructed a DAG illustrating the corresponding causal pathways (Fig. 2). Aspiration was the potential mediator variable, and thus, it was incorporated into the multivariate models of penetration and residue to assess whether the associations between penetration, residue, and pneumonia ceased.

In our analysis of the effect of penetration, residue, and aspiration on pneumonia occurrence, we considered all subjects exhibiting the respective alteration, regardless of the presence of aspiration. For example, in the analysis of penetration, we incorporated subjects with both penetration and aspiration, as well as those with penetration alone.

Incorporating the aspiration variable into the multivariate models aimed to assess whether penetration or residue necessitated the presence of aspiration to increase the risk of pneumonia. This involved controlling for aspiration, effectively isolating the effect of penetration or residue (i.e., excluding the influence of aspiration) on pneumonia occurrence. If the association of penetration or residue with pneumonia occurrence were independent of the presence of aspiration, such an association would remain statistically significant even after introducing the aspiration variable into the corresponding models.

In our study, we analyzed aspiration, penetration, and pharyngeal residue as dichotomous variables. This approach allowed us to explore independent causal pathways in the multivariate analysis, which would have been challenging with ordinal scales. Moreover, incorporating ordinal, non-normally distributed variables into multivariable models presents statistical challenges that could interfere with achieving our main objectives.

The sample size calculation was performed with data from a prior retrospective study [43]. A total sample size of 148 subjects was calculated estimating a 20% annual incidence of pneumonia in the exposed cohort, a 4% annual incidence of pneumonia in the unexposed cohort, a 12% mean incidence (cohort exposed and unexposed combined), and a follow-up of 5 years (confidence level: 95%; power: 80%; including 10 predictor variables in the multivariate logistic regression model). The software used was Microsoft Excel 2007, and Stata SE, version 15.

## Results

A total of 148 patients were recruited from the comprehensive rehabilitation program at the University of La Sabana clinic and followed up for 5 years. The median age was 51 years with 57% being male. The clinical diagnoses with the highest prevalence were cerebrovascular disease (34%), trauma (18%), and neurodegenerative diseases (13%). The most common comorbidities were COPD (11%), heart failure (7%), and diabetes (7%). Approximately 60% of the population used antacids and 18% received psychiatric treatment, as shown in Table 1. The detailed characteristics of patients with and without aspiration may be found in the supplementary appendix (Table 1 of supplementary appendix).

**Table 1:** General characteristics of the cohort subjects

Characteristics	Sample
Nº. (%)	n = 148 (100)
Age in years, median (IQR)	51 (32–70)
Sex—no. (%)	
Male	85 (57)
Swallowing disorders—no. (%)	
Residue	107 (72)
Penetration	98 (66)
Aspiration	76 (51)
Penetration and aspiration	72 (49)
Residue and aspiration	71 (48)
Pneumonia—no. (%)	
Patients who presented at least one pneumonia—no. (%)	49 (33)
Total number of pneumonias in all patients—no	105
Underlying disease—no. (%)	
Cerebrovascular disease	50 (34)
Brain trauma	27 (18)
Neurodegenerative diseases	19 (13)
Tumor	10 (7)
Hypoxic encephalopathy	6 (4)
Gastroesophageal reflux	6 (4)
Muscular dystrophy	5 (3)
CNS infections (including sequels)	5 (3)
Others	5 (3)
Neuropathies, radiculopathies, and myelopathies	4 (3)
Comorbidities—no. (%)	
COPD	17 (11)
Congestive heart failure	10 (7)
Diabetes	10 (7)
Immunosuppression	7 (5)
Use of antacids—no. (%)	
Antacids	89 (60)
Psychiatric treatment—no. (%)	
Antipsychotics	27 (18)
Swallowing disorders—no. (%)	
Residue	107 (72)
Penetration	98 (66)
Aspiration	76 (51)
Smoking—no. (%)	
Currently smoke	5 (3)
Mouth wash^a^—no. (%)	
Two or more times per day	132 (89)
Alcohol consumption^b^—no. (%)	
High alcohol consumption	13 (9)
Socioeconomic level^c^—no. (%)	
Socioeconomic level low	29 (20)
Use of corticosteroids—no. (%)	
Inhaled corticosteroid	21 (14)
Systemic corticosteroid	12 (8)

*IQR* interquartile range (25th percentile to 75th percentile)

^a^Mouth washing: refers to the number of mouth washings per day

^b^High alcohol consumption: in men frequency greater than or equal to 8 drinks on one occasion at least once a month, women: frequency greater than or equal to 6 drinks on one occasion at least once a month or for both sexes’ greater consumption or equal to 3 alcoholic drinks daily

^c^Socioeconomic level low: those classified as 1 and 2 (see materials and methods)

For additional information on underlying diseases and comorbidities, please refer to the supplementary appendix

In the Binomial regression model, the risk of pneumonia was significantly higher when the following FEES findings were present: aspiration with an adjusted IR of 26.6 cases of pneumonia per 100 people-years (*P* < 0.001), penetration with an adjusted IR of 19.7 cases of pneumonia per 100 people-years (*P* < 0.001), and residue with an adjusted IR of 18.1 cases of pneumonia per 100 people-years (*P* < 0.001).

The bivariate analysis (Table 2) was conducted using negative binomial regression, as the variables were overdispersed, meaning that the standard deviation of the response variable was greater than its mean. Significant associations were found with the variables of aspiration, penetration, and residue (*P* < 0.001), and a significant trend was observed with the use of antiacids.

**Table 2:** Bivariate analysis between pneumonia and possible risk factors

Dependent variable	Independent variable	IRR	*P* value
Number of pneumonias since the event	Aspiration	7.24	< 0.001
	Penetration	7.70	< 0.001
	Residue	5.84	< 0.001
	Age at time of event	0.99	0. 149
	Current smoking	0.62	0.661
	Sex	1.31	0.459
	Socioeconomic level low^a^	0.76	0.582
	Mouthwash^b^ (more than twice a day)	0.51	0.306
	High alcohol consumption^c^	0.90	0.878
	COPD	1.12	0.839
	Asthma	0.00	0.999
	Other respiratory diseases	1.06	0.815
	Diabetes	0.92	0.906
	Congestive heart failure	0.61	0.526
	Immunosuppression	0.16	0.148
	Inhaled corticosteroid	1.40	0.515
	Systemic corticosteroid	0.39	0.229
	Antacids	2.01	0.070
	Proton-pump inhibitors	1.69	0.165

^a^Socioeconomic level low: those classified as 1 and 2

^b^Mouth washing: refers to the number of mouth washings per day

^c^High alcohol consumption: in men frequency greater than or equal to 8 drinks on one occasion at least once a month, women: frequency greater than or equal to 6 drinks on one occasion at least once a month or, for both sex, greater consumption or equal to 3 alcoholic drinks daily

For the multivariate analysis to control for confounding bias, we selected variables that could be potentially associated with the studied outcome (pneumonia) and the exposure (FEES alterations) based on both clinical relevance and a *P* < 0.25. The clinical relevance was determined by biological plausibility based on previous studies and DAG (Fig. 1).

The relative risk of developing pneumonia increased by 7.25 times (*P* < 0.001) in patients who had aspiration, 7.85 (*P* < 0.001) in those who had penetration, and 6.24 times (*P* < 0.001) in those who had pharyngeal residue, after adjusting for possible confounding variables (Tables 3, 4, 5).

**Table 3:** Multivariate binomial regression model for aspiration

Number of pneumonias since the event	IRR	95% Conf. Interval		*P*
Aspiration	7.25	3.50	14.98	0.001
Age at time of event	0.99	0.98	1.01	0.399
Antacids	1.46	0.72	2.96	0.300
Sex	1.18	0.60	2.30	0.632
Mouthwash^a^ (more than twice a day)	0.40	0.12	1.33	0.135
Socioeconomic level low^b^	1.06	0.44	2.60	0.894
Immunosuppression	0.27	0.03	2.44	0.241

^a^Mouthwash refers to the number of mouthwashes per day

^b^Socioeconomic level low: those classified as 1 and 2

**Table 4:** Multivariate binomial regression model for penetration

Number of pneumonias since the event	IRR	95% Conf. Interval		*P*
Penetration	7.85	3.34	18.47	0.001
Age at time of event	0.99	0.98	1.00	0.122
Antacids	1.84	0.92	3.66	0.083
Mouthwash^a^ (more than twice a day)	0.40	0.13	1.27	0.119
Immunosuppression	0.16	0.02	1.72	0.131

^a^Mouthwash: refers to the number of mouth washings per day

**Table 5:** Multivariate binomial regression model for residue

Number of pneumonias since the event	IRR	95% Conf. Interval		*P*
Residue	6.24	2.58	15.09	0.001
Age at time of event	0.99	0.98	1.00	0.109
Antiacids	1.68	0.83	3.42	0.151
Mouthwash^a^ (more than twice a day)	0.37	0.11	1.20	0.097
Immunosuppression	0.14	0.01	1.52	0.107

^a^Mouthwash: refers to the number of mouth washings per day

To explore if the association between penetration and residue with pneumonia was independent or depended on the association of these abnormalities with aspiration, the aspiration variable was introduced into the multivariate models of penetration and residue (see corresponding DAGs in Fig. 2). After doing this, the association between penetration and residue with pneumonia disappeared, however, the association between aspiration and pneumonia persisted, suggesting that the association between penetration and residue with pneumonia is not independent, but rather depends on whether these abnormalities cause aspiration, which would ultimately lead to pneumonia (Tables 6, 7).

**Table 6:** Effect of adjusting for aspiration in the penetration binomial regression model

Number of pneumonias since the event	IRR	95% Conf. Interval		*P*
Penetration	2.63	0.77	9.04	0.124
Aspiration	3.68	1.27	10.62	0.016
Age at time of event	0.99	0.98	1.01	0.237
Antiacids	1.56	0.78	3.12	0.204
Mouthwash^a^ (more than twice a day)	0.40	0.13	1.25	0.115
Immunosuppression	0.24	0.03	2.24	0.212

^a^Mouthwash: refers to the number of mouth washings per day

**Table 7:** Effect of adjusting for aspiration in the residue binomial regression model

Number of pneumonias since the event	IRR	95% Conf. Interval		*P*
Residue	2.01	0.67	6.00	0.212
Aspiration	4.85	1.95	12.08	0.001
Age at time of event	0.99	0.98	1.01	0.266
Antiacids	1.49	0.75	2.97	0.258
Mouthwash^a^ (more than twice a day)	0.38	0.12	1.20	0.099
Immunosuppression	0.23	0.03	2.16	0.201

^a^Mouthwash: refers to the number of mouth washings per day

## Discussion

In this study, performed in a cohort of patients with various causes of functional oropharyngeal dysphagia, representative of many swallowing services of tertiary care centers, we found an increased risk of pneumonia in the presence of aspiration, penetration, or residue with any consistency of food in the FEES. However, only aspiration was independently associated with pneumonia because the association of penetration or residue with pneumonia disappears adjusting with aspiration. In addition, we observed that the frequency of mouth washing tended to be associated to a lower risk of pneumonia, meaning that it could be a protective factor for the development of pneumonia, since it possibly decreases oral colonization as reported by Aiemyen et al. [44].

We found a 7.2 times increased risk of pneumonia in patients with detected aspiration in the FEES, similar to those reported by Holas et al. [18] and Pikus et al. [20] in studies performed by VFSS. Previous studies with FEES found an increased risk of pneumonia without reaching statistical significance, except in the study by Takahashi et al., [45] where they found a significant increase in the risk of pneumonia in patients with aspiration of saliva; however, aspiration of food was not associated with pneumonia in that study. The fact that all of these previous studies consistently showed an annual incidence of pneumonia higher than 20% in subjects with oropharyngeal dysphagia when the incidence of pneumonia reported for the general population is below 1.5% [46, 47], and that pooling these studies’ results in a recent meta-analysis [24] reached statistical significance, suggests that the absence of statistical significance in such original publications was probably related to low statistical power due to small sample sizes, or insufficient numbers of patients without aspiration, and not to a real lack of association between aspiration and pneumonia.

Prior research by Pikus et al. [20] and Gurber et al. [48] found a significant increase in pneumonia risk among patients with penetration observed in VFSS, but they did not investigate the effect of adjusting for aspiration in this association. Our findings indicate that the relationship between penetration and pneumonia could be contingent upon whether penetration leads to aspiration rather than penetration itself causing pneumonia. Conversely, no previous studies have demonstrated a link between pneumonia and pharyngeal residue detected in VFSS or FEES. Nevertheless, evidence suggests an elevated risk of aspiration due to pharyngeal residue in both VFSS [49, 50] and FEES [51–53]. Our findings point to that the association between residue and pneumonia disappears when adjusting for aspiration, indicating that the relationship may hinge on whether pharyngeal residue leads to aspiration, mirroring the situation observed with penetration.

This study has the limitations of a cohort study; that is, it is exposed to biases and confounding variables which limit its ability to infer definitive causal relationships. However, the fact that this is one of the largest cohort studies that has been done evaluating the risk of pneumonia associated with alterations of swallowing safety detected in FEES, in terms of number of patients and time of follow-up, and that we analyzed outcome (pneumonia) as IR instead of cumulative incidence, as recommended when a subject may have more than one event (pneumonia) [54], helped to decrease the limitations related to statistical power. Furthermore, we took several measures to reduce biases (see the methods section), which may have helped to obtain more accurate risk estimates.

The diagnosis of pneumonia can be a challenge for any study because the diagnostic criteria have no high specificity or sensitivity [29, 33]. However, the diagnosis based on clinical criteria supported by imaging has been widely accepted for more than 20 years, particularly in the centers where these patients were treated; furthermore, it is consistent with the diagnoses used in other studies on dysphagia complications [20, 29, 33, 55]. In addition, the doctors who made the pneumonia diagnoses were unaware of the study and the hypothesis thereof, which minimized the risk of differential misclassification. The risk of non-differential misclassification cannot be completely ruled out in our study and is common in any study that uses a pneumonia outcome, due to the diagnostic difficulties mentioned above, but this type of bias often results in an underestimation of the relative risk (RR) instead of an overestimation, so it does not invalidate a significant RR [56].

## Conclusions

Our study presents evidence about the independent significant association between aspiration detected in FEES and the development of pneumonia. In addition, it showed that alterations in the oral and pharyngeal phases of swallowing, without the presence of aspiration, did not significantly increase the risk of developing pneumonia. Patients in whom aspiration is observed during FEES should receive interventions aimed at reducing the risk of pneumonia, including dietary modifications to avoid oral feeding with the food consistency that caused aspiration, proper oral hygiene, influenza and pneumococcal vaccination, avoidance of medications that can increase the risk of pneumonia, and, when clinically indicated (e.g. aspiration of more than two food consistencies) be fed by an alternative route to the oral one [33, 57].

## Supplementary Information

Below is the link to the electronic supplementary material.

Supplementary file1 (DOCX 6634 KB)

## Data Availability

The datasets generated during and/or analysed during the current study are available from the corresponding author on reasonable request.
