# Supplementary material for: Fiberoptic Endoscopy Evaluation of Swallowing (FEES) Findings Associated with High Pneumonia Risk in a Cohort of Patients at Risk of Dysphagia
Source: Dysphagia. 2024 Jul 3;40(1):282–91. doi: 10.1007/s00455-024-10727-w (PMC11762443; doi:10.1007/s00455-024-10727-w)
Supplement: Supplementary file 1 — Supplementary file1 (DOCX 6634 KB) [file 455_2024_10727_MOESM1_ESM.docx]

**APPENDIX**

**Clinical evaluation of swallowing**

Patients underwent a standardized clinical evaluation of swallowing looking for signs and symptoms of oropharyngeal dysphagia such as alteration in food recognition, hypersalivation, food residue in the oral cavity, a feeling of adherence of food to the oral cavity, nasal regurgitation, wet voice after swallowing or dysarthria, cough after swallowing, choking, difficulty in starting to swallow, delay or absence of the swallowing reflex, impaired or absence of the gag reflexes and cough, evidence of food in a tracheostomy, respiratory complications (bronchorrhoea, bronchospasm, pneumonia, and pulmonary fibrosis), presence or history of gastrostomy, and impairment of oropharyngeal muscles and other phonoarticulatory organs (jaw muscles, lips, cheeks, tongue, palate, pharynx, and larynx) [1,2]. The examination also included an assessment of the functionality of the head and neck muscles, identification of whether alterations affected the phonoarticulatory organs or other head and neck muscles, observation of any possible dysarthria or apraxia of swallowing by evaluating different food consistencies, and observation of the presence of food residues in the oral cavity.

Subsequently, a volume and viscosity test was performed in which each patient received food boluses of 5 to 20 mL with differing food consistencies, starting with thick liquids (nectar) and soft solids (puree), and then solids (graham cracker) and clear liquids. If the patient had coughing, hoarseness, choking, or a wet voice with one of the consistencies, the test was considered positive[1,3].

The food is prepared with commercial yogurt made from cow’s milk, which has a thick fluid consistency, it is mixed with water to obtain a thin-liquid consistency or with food thickener (modified cornstarch, Spezante^®^; Boydorr Nutrition, Chia, Colombia) to obtain a semi-solid (puree) consistency. A Graham cracker provides the needed solid-food consistency for the test. During the FEES, all the foods were colored green with food coloring in order to improve their endoscopic visibility, but green coloring was not added for the clinical test of swallowing (Figure 1).

The subject receives at least three boluses (here “bolus” refers to the volume of food that the subject receives in his mouth) of every food consistency. The SPL gives the food to the subject using a spoon or, in the case of liquids, using a straw and a cup. The volume of bolus is progressively increased from 2.5 cc to 10 cc.


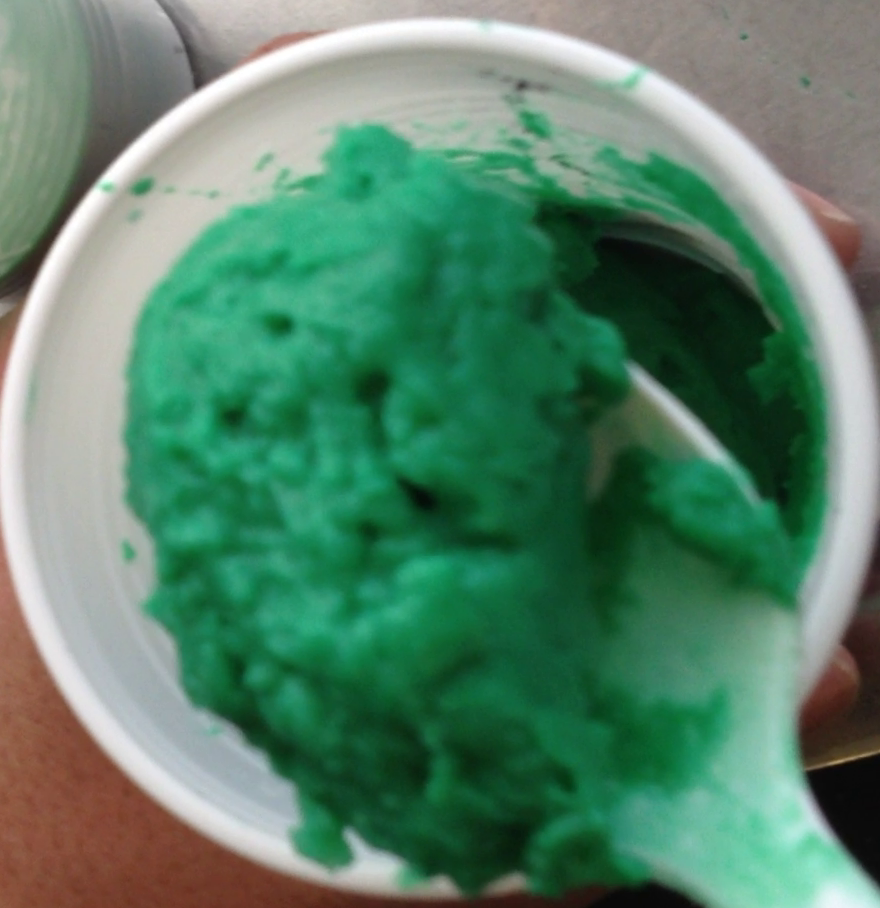

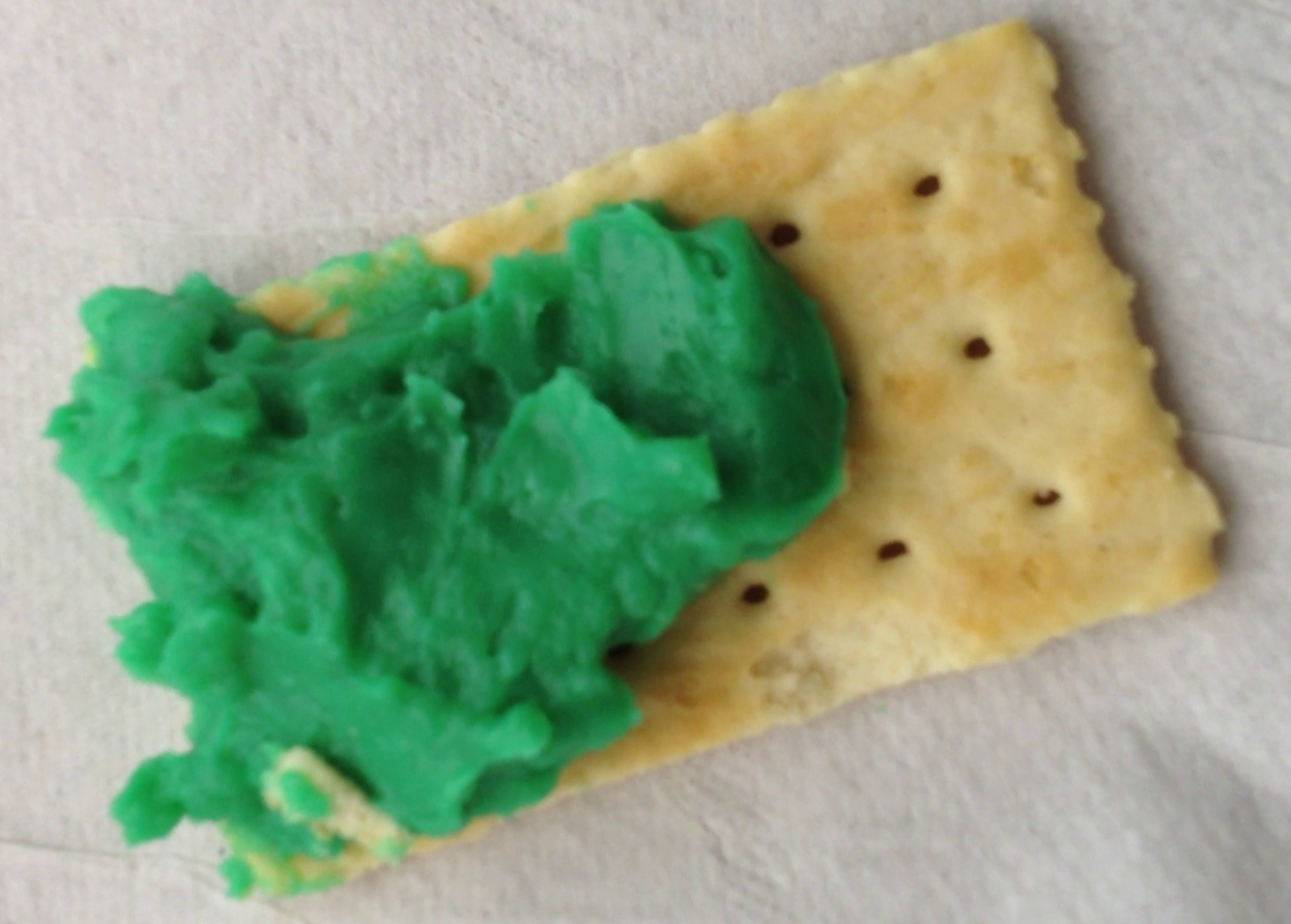


a) b)


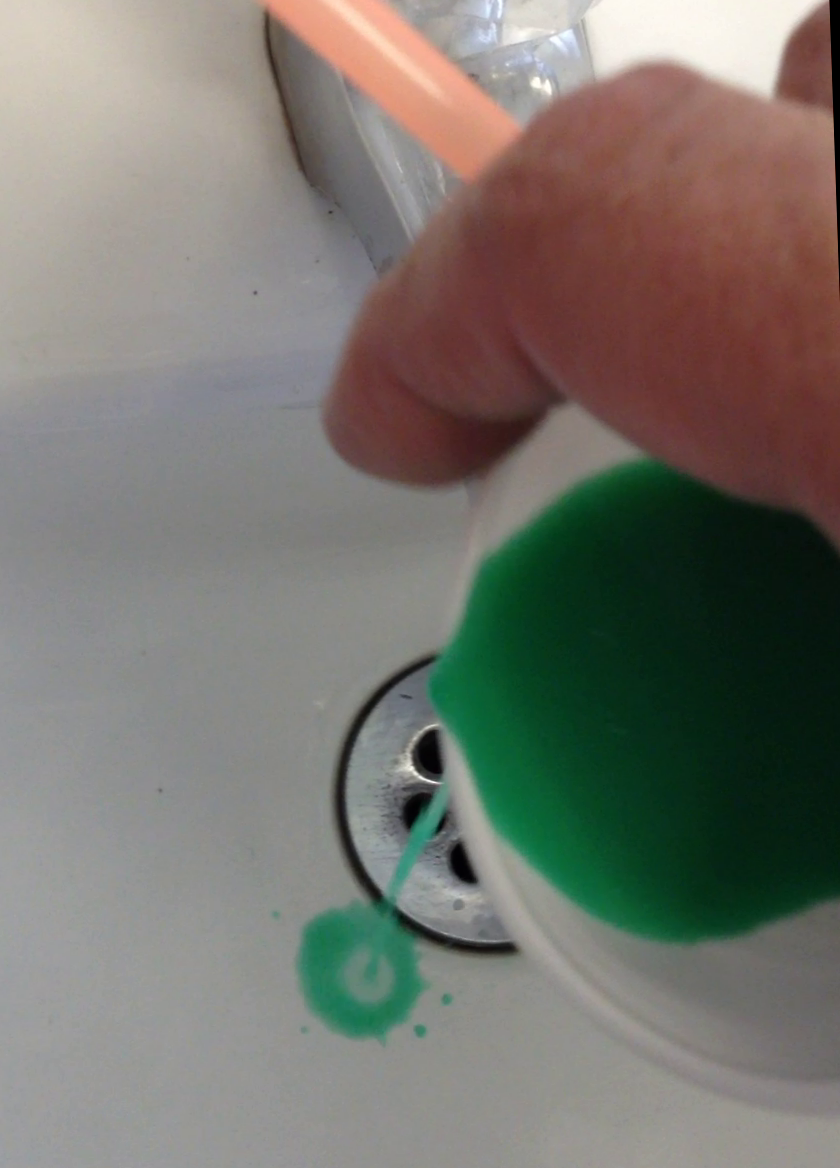

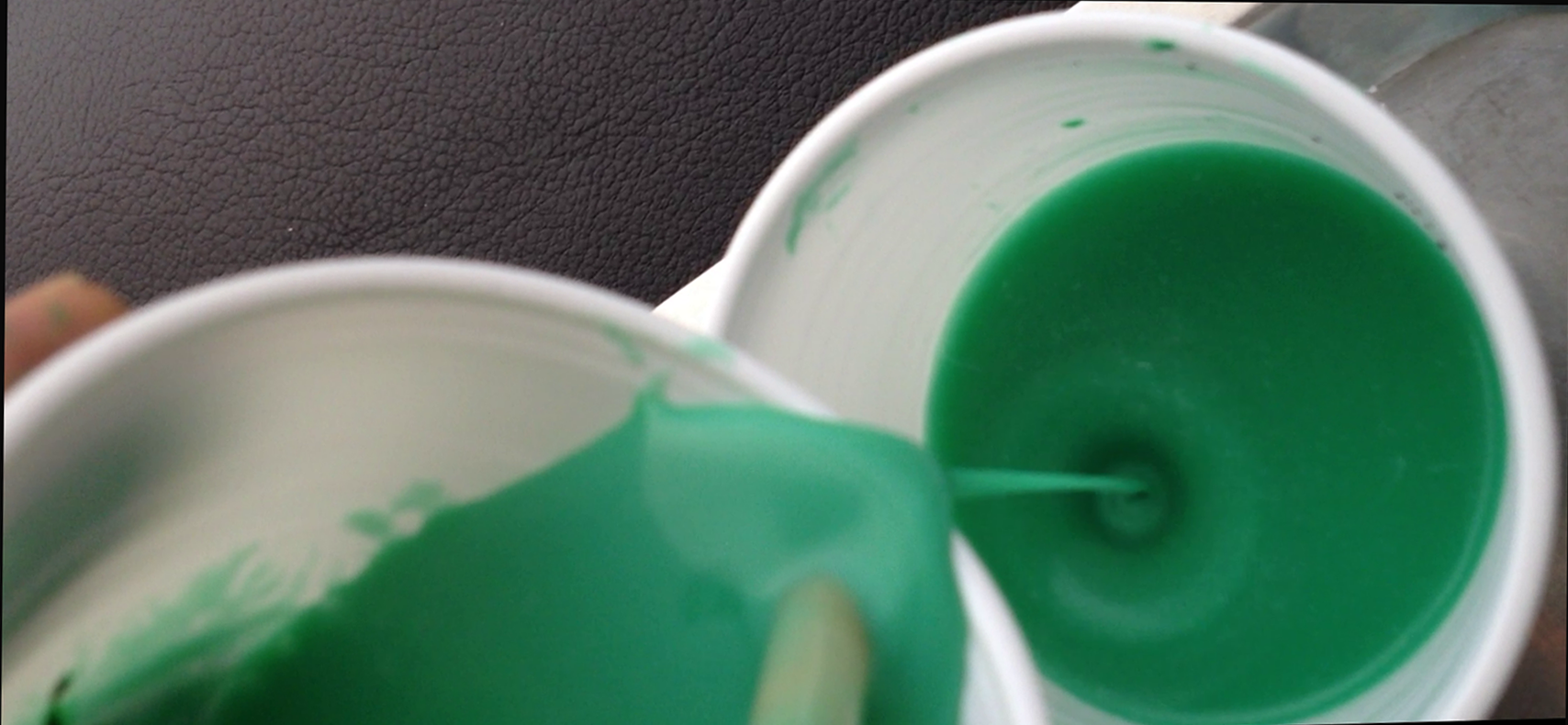


c) d)

**Figure 1. Food consistencies used for the endoscopic evaluation of swallowing:** a) Semi-solid (puree); b) Solid (Graham cracker covered with green puree for coloring); c) Thin liquid; d) Thick liquid. All of the foods were colored green with food coloring in order to improve their endoscopic visibility.

**Bias control**

To reduce selection bias, new patients at risk of oropharyngeal dysphagia admitted to our institution during the study period were recruited consecutively. Likewise, to control observer bias and to prevent information loss, standardized forms for obtaining medical histories, clinical evaluations of swallowing, FEES, and follow-ups were used. Telephone and face-to-face follow-ups were performed by people blinded to the results of the FEES and the clinical swallowing evaluation. The staff who collected the data, performed follow-ups, and identified data in medical records were trained by our research team.

**Supplementary appendix Table 1.** General characteristics of the exposed cohort

|  | **Exposure** | | |
| --- | --- | --- | --- |
| **Characteristics** | **Sample** | **Aspiration** | **Non aspiration** |
| **Nº. (%)** | **n=148 (100)** | **n=76(51)** | **n=72(49)** |
| Age in years, median (IQR) | 51 (32 - 70) | 57 (31 - 71) | 48 (33 - 65) |
| **Sex - no. (%)** |  |  |  |
| Male | 85 (57) | 50 (66) | 35 (49) |
| **Pneumonia – no. (%)** |  |  |  |
| Patients who presented at least one pneumonia – no. (%) | 49 (33) | 36 (47) | 13 (18) |
| Total number of pneumonias in all patients – no. | 105 | 90 | 15 |
| **Underlying disease - no. (%)** |  |  |  |
| Cerebrovascular disease | 50 (34) | 26 (34) | 24 (33) |
| Trauma | 27 (18) | 10 (13) | 27 (38) |
| Neurodegenerative diseases | 19 (13) | 11 (14) | 8 (11) |
| Tumor | 10 (7) | 6 (8) | 4 (6) |
| Hypoxic encephalopathy | 6 (4) | 5 (7) | 1 (1) |
| Gastroesophageal reflux | 6 (4) | 1 (1) | 5 (7) |
| Muscular dystrophy | 5 (3) | 3 (4) | 2 (3) |
| Infections (including sequelae) | 5 (3) | 2 (3) | 3 (4) |
| Others. | 5 (3) | 5 (7) |  |
| Neuropathies, radiculopathies and myelopathies. | 4 (3) | 2 (3) | 2 (3) |
| Connective tissue disorders | 3 (2) | 1 (1) | 2 (3) |
| Surgeries in the pharynx or larynx | 2 (1) | 1 (1) | 1 (1) |
| HIV | 1 (1) | 1 (1) |  |
| Chronic kidney disease | 1 (1) |  | 1 (1) |
| **Comorbidities - no. (%)** |  |  |  |
| COPD | 17 (11) | 12 (16) | 5 (7) |
| Congestive heart failure | 10 (7) | 5 (7) | 5 (7) |
| Diabetes | 10 (7) | 5 (7) | 5 (7) |
| Immunosuppression | 7 (5) | 3 (4) | 4 (6) |
| Asthma | 5 (3) | 3 (4) | 2 (3) |
| Cirrhosis | 2 (1) | 1 (1) | 1 (1) |
| **Use of antacids - no. (%)** |  |  |  |
| Antacids | 89 (60) | 48 (63) | 41 (57) |
| **Psychiatric treatment - no. (%)** |  |  |  |
| Antipsychotics | 27 (18) | 11 (14) | 16 (22) |
| **Smoking - no. (%)** |  |  |  |
| Currently smoke | 5 (3) | 2 (3) | 3 (4) |
| **Mouth wash - no. (%)** |  |  |  |
| Two or more times per day | 132 (89) | 66 (87) | 66 (92) |
| **Alcohol consumption - no. (%)** |  |  |  |
| High alcohol consumption | 13 (9) | 6 (8) | 7 (10) |
| **Socioeconomic level - no. (%)** |  |  |  |
| Socioeconomic level low | 29 (20) | 18 (24) | 11 (15) |
| **Use of corticosteroids - no. (%)** |  |  |  |
| Inhaled corticosteroid | 21 (14) | 14 (18) | 7 (10) |
| Systemic corticosteroid | 12 (8) | 6 (8) | 6 (8) |

***High alcohol consumption:** in men frequency greater than or equal to 8 drinks on one occasion at least once a month, women: frequency greater than or equal to 6 drinks on one occasion at least once a month or for both sexes greater consumption or equal to 3 alcoholic drinks daily. ***Mouth washing:** refers to the number of mouth washings per day. ***IQR**: interquartile range (25th percentile – 75th percentile). ***Socioeconomic level low**: those classified as 1 and 2.

**References**

1. Pere Clavé, Viridiana Arreola, Mercedes Velasco, Miquel Quer, Josep Maria Castellví, Jordi Almirall, et al. Diagnóstico y tratamiento de la disfagia orofaríngea funcional. Aspectos de interés para el cirujano digestivo. Cir Esp. 2007;62–76.
2. Horna-Castiñeiras J, Fernández-Bermejo E, Raboso García-Baquero E. Valoración y manejo clínico del paciente con disfagia. Formación médica continuada en atención primaria. 2008;78–91.
3. M^a^ Mercedes Velasco, Viridiana Arreola, Pere Clavé, Carolina Puiggrós. Abordaje clínico de la disfagiaorofaríngea: diagnóstico y tratamiento. Nutrición Clínica en Medicina. 2007;I:174–202.
